# Nurses and doctors ‘s awareness and knowledge towards treatment and care of cervical cancer patients: a cross-sectional study

**DOI:** 10.1186/s12912-023-01522-3

**Published:** 2023-10-18

**Authors:** Zhen Li, Sinuo Chen, Ishrat Mahjabeen, Rabia Shafique

**Affiliations:** 1 Department of Cancer Center Day Ward, Henan Provincial Kev Medicine Laboratory of Nursing, Henan Provincial People’s Hospital, Zhengzhou City, Henan 450003 China; 2 People’s Hospital of Zhengzhou University, Zhengzhou City, Henan 450003 China; 3 People’s Hospital of Henan University, Zhengzhou City, Henan 450003 China; 4 College of Nursing and Health, School of Nursing and Health, Henan University, Kaifeng City, Henan Province 475004 China; 5 Cancer Genetics and Epigenetics Research Group, Department of Biosciences, COMSATS University Islamabad, Islamabad City, Pakistan

**Keywords:** Cervical cancer, Patient’s care, Nursing care, Qualitative study, Healthcare professionals, HPV vaccination, Treatment modalities

## Abstract

**Aim:**

The present study aimed to investigate healthcare professionals’ perceptions and experiences in caring for cervical cancer patients. The present study was also designed to assess the healthcare professionals’ attitudes toward cervical cancer screening and its prevention.

**Methods:**

A cross-sectional quantitative descriptive study was conducted, and 540 participants (240 nurses and 300 doctors), from different hospitals of Pakistan have been selected and interviewed.

**Results:**

Data was collected using structured questionnaires and SPSS was used to statistically analyze the data. Participants in the present study are questioned with respect to age, gender, and work experience. The mean age of the participants is 35 years. Among them, 41% of participants are < 35 years of age and 59% are > 35 years of age. In the case of gender, 22% of participants are males and 78% are females. 47% of the participants have work experience < 20 years and 53% have work experience > 20 years. Data from the present study showed that most of the nurses are less educated (basic education of middle and matric degree) with a simple diploma in nursing and midwifery. Nurses and doctors do not have any knowledge/experience of the patient’s psychological counselling. Participants are also questioned with respect to HPV vaccination, 39% of nurses and 62% of doctors are vaccinated. The difference in vaccination frequency of participants was observed as statistically significant (p < 0.0001). In the case of treatment modalities, doctors have statistically more knowledge about the pap smear (p < 0.0001), cervical biopsy (p < 0.0001), colposcopy (p < 0.0001), and visual application after acetic acid application (p < 0.0001) compared to nurses. Data analysis showed that Pap smear was performed significantly higher in married females compared to unmarried (p < 0.0001).

**Conclusion:**

our study provides a comprehensive and in-depth perspective of the nurses and doctors for cervical cancer patients. Cervical cancer prevalence is increasing due to inadequate knowledge and awareness among healthcare professionals. Improvement can be brought about by the regular use of treatment modalities in unmarried females also.

## Introduction

Cervical cancer is one of the most common gynecological tumors affecting women. It is the fourth most diagnosed cancer in women and also the fourth leading cause of cancer-related deaths [1]. According to GLOBOCAN 2020 cancer statistics, 604,000 new cases and 342,000 deaths occurred globally due to cervical cancer [1]. Approximately 85% of deaths occur in women living in low-income countries because of a lack of awareness and limited access to health services [2]. This lack of awareness and health services in low-income countries may results in, increased burden/complications of cervical cancer in future [2]. In Pakistan, cervical cancer is the third most common cancer among women with a reported incidence rate of 5.98% [3].

Cancer of cervix is a rapid growth of anomalous cells of cervix [4]. Contributory factors that make women vulnerable to cervical cancer include infection with sexually transmitted viruses such as HPV, long term use of oral contraceptives, multiple pregnancies, tobacco smoking, immunosuppression, and low socio-economic status [4, 5]. HPV a sexually transmitted infection is a key causative agent found in almost every case of cervical cancer [6]. HPV genotypes, HPV16 and HPV18 are responsible for 70% of cervical cancer [7]. Among these genotypes, HPV16 is considered as the major risk factor in the development of invasive cervical carcinoma [7]. HPV vaccines are created using L1 virus like particles of CPS component of HPV subtypes and it generate the antibody titer that is enough to neutralize the diverse strains of HPV and ultimately triggers the humoral immune response against illness and dysplastic lesions [8, 9]. Farhath et al., (2013) has reported that HPV vaccination is effective against the HPV16 and HPV18 to prevent its infectious rate and spread effectively [10]. Two HPV vaccines are commercially available. One is Cervarix, targets the two HPV subtypes and second is Gardasil, targets four HPV subtypes. HPV vaccine is more effective, when administered before the sexual activation and exposure to HPV [11, 12]. Hanson et al., [2015] has reported that administration of HPV vaccination before the age of adolescence found associated with increased HPV antibody titer and strong immunoreactivity compared with young adult [13]. HPV vaccine provides the long term protection against the infection caused by high-risk and low-risk HPV strains and minimal level of cross protection [14]. Reason behind of not getting HPV vaccination before the age of adolescence are (i) parents do not understand the importance of HPV vaccine and its impact on the cancer prevention. (ii) lack of HPV vaccination recommendations from the health care professionals [13, 14].

Cervical cancer is a preventable and curable disease because of availability of highly effective HPV vaccine and screening measures, a substantial decrease in cervical cancer cases have been observed in western countries [15]. This is mainly attributed to early screening, awareness, and treatment of pre-invasive cervical lesions [16]. However, cervical cancer is a major health concern/problem in low-income countries where 1/3rd of women diagnosed with cervical carcinoma eventually die [17]. About 50–90% of women in Asia, who diagnosed or die with cervical carcinoma have never been screened [17, 18].

Cervical cancer remains a significant public health issue worldwide, particularly in India and the SAARC nations (Afghanistan, Bangladesh, Bhutan, India, Maldives, Myanmar, Nepal, Pakistan, and Sri Lanka) [6, 19]. Cervical cancer burden estimate for Pakistan has been limited and regional. WHO disease burden estimate from Pakistan is based on the data from the neighboring countries and limited data from Pakistan [20]. Pakistan has an estimated cervical cancer burden that exceeds the WHO target [20]. According to data retrieved from major hospitals of Pakistan, 7.06 (95% CI = 5.98–10.01) is age standardized incidence rates (ASIR) for cervical cancer with an estimated new case of 6166 per year [20]. The exact situation of cervical cancer is not known in Pakistan because of social stigma and its ignorance in term of screening and prevention [21]. It has been reported that only 5% of women in Pakistan are aware of cervical cancer screening furthermore only 2.5% had Pap smear test once in a lifetime [20]. Lack of screening practices, poor knowledge about disease, unavailability of Pap smear test and lack of follow-up increases the disease burden in Pakistan [21].

Nurses and doctors are important health professionals who can effectively guide women regarding cervical cancer screening and treatment, thereby immensely contributing to reduction of cervical cancer morbidity and mortality [15] Medical professionals have greater impact on general population so they can effectively improve the screening practices and tackle the situation. In case of Pakistan, one of reason of increased cervical cancer burden is lack of knowledge/awareness of cervical cancer and inadequate knowledge in nursing care. Healthcare professionals’ lack of knowledge and misconceptions have resulted in suboptimal care due to insufficient knowledge given to the patients [22]. The misconceptions and inadequate knowledge about disease hinders nursing care being given to cancer patients by oncological health professionals. Experienced nurses and doctors with sufficient knowledge about disease have more positive attitude toward their patients than unexperienced ones [22]. Therefore, nurses should have sound knowledge about cervical cancer so they can provide optimal care and information to patients. Most of the studies have been published on the evaluation of genetic/expression variation of different genes/pathways in cervical carcinogenesis [23–27]. Limited number of studies have been published with respect the cervical cancer and cervical cancer screening awareness among healthcare professionals [28–30]. In Pakistan no study has been reported yet on nurses and doctors’ awareness for cervical cancer prevention. This gap in knowledge can be root cause of the uncontrolled disease burden/mortality in Pakistan. So, there is need to first determine healthcare professional s’ level of awareness about cervical cancer patients care and handling. Second, determination of healthcare professional s’ knowledge for the cervical cancer screening test and preventions is also needed to be quantified. This study was designed to investigate the knowledge of cervical cancer, risk factors and preventive measures among the healthcare professionals (doctors and nurses) working in different hospitals of Pakistan. Furthermore, to assess the knowledge of screening practices of female health professionals/ nurses toward cervical cancer.

## Methodology

### Study design

A cross-sectional quantitative descriptive study was conducted in the six main hospitals in north Pakistan including the Pakistan Institute of Medical Sciences (PIMS, Islamabad), Holy Family Hospital (Rawalpindi), Benazir Bhutto Hospital (Rawalpindi), Central Hospital (Rawalpindi), Social Security Hospital (Rawalpindi) and Polyclinic Hospital (Islamabad).

Nurses and doctors working in the following hospitals were selected as study population. We visited these hospitals and explained the purpose of the study, its aim and objectives to health professionals and nurses and asked for their cooperation. Participation was totally voluntary and written consent was taken from each participant prior to sampling. 100–150 participants were included from each hospital. There was no financial/material incentive provided for participation. The confidentiality and anonymity of participants was ensured, and respondents have full authority to withdraw from study at any point. Ethical approval was taken from the ethical review committees of both COMSATS university Islamabad, Pakistan, and participating hospitals.

### Sample size determination

Single population proportion formula (formula used to compare the proportions of responses with proportion of population from which data is drawn) with 95% CI, 5% precision was used to determine the sample size. Responses collected from the heathcare professionals are the type of samples in the present study. No regional study was conducted on this topic previously so an assumed knowledge and preventive practices of cervical cancer 50% was used to attain maximum sample size. The calculated sample size came out was 210 that increased to 221 by adding 10% allowance for non-responses and incomplete questionnaires. However, the total questionnaires came after survey were 540 (300 doctors and 240 nurses). Inclusion criteria includes nurses having 3 years of experience in Oncology and Outpatient Departments. Any participant who did not provide written consent or give incomplete questionnaire was excluded from the study.

### Data collection and analysis

The standardized close-ended structured questionnaire was designed and verified by experts for the present study. The study subjects were not required to write descriptive answers, instead they just needed to tick/cross in appropriate boxes given for each question. The questionnaire consisted of a section on sociodemographic profile of subjects which consist of name, age, gender, educational status, marital status, religion, economic status, number of pregnancies, years of employment and department of employment. Second section is about their personal status of disease such as previous diagnosis of cervical cancer, family history of disease, vaccination with HPV. Last section is about knowledge regarding cervical cancer and practices and experiences in cancer wards such as terrible prognosis of disease, prejudice of cervical cancer, sympathy toward patients, psychological counseling of patients and awareness about cervical cancer prevention in masses and role of health professionals.

The data obtained was entered and analyzed using statistical package for social sciences (SPSS) version 6.0 software tool. Percentages and other analysis were calculated for all variables and compatible tables and graphs were computed. Chi square test was used with p value ≤ 0.05 was considered as statistically significant.

## Results

**Fig. 1:**
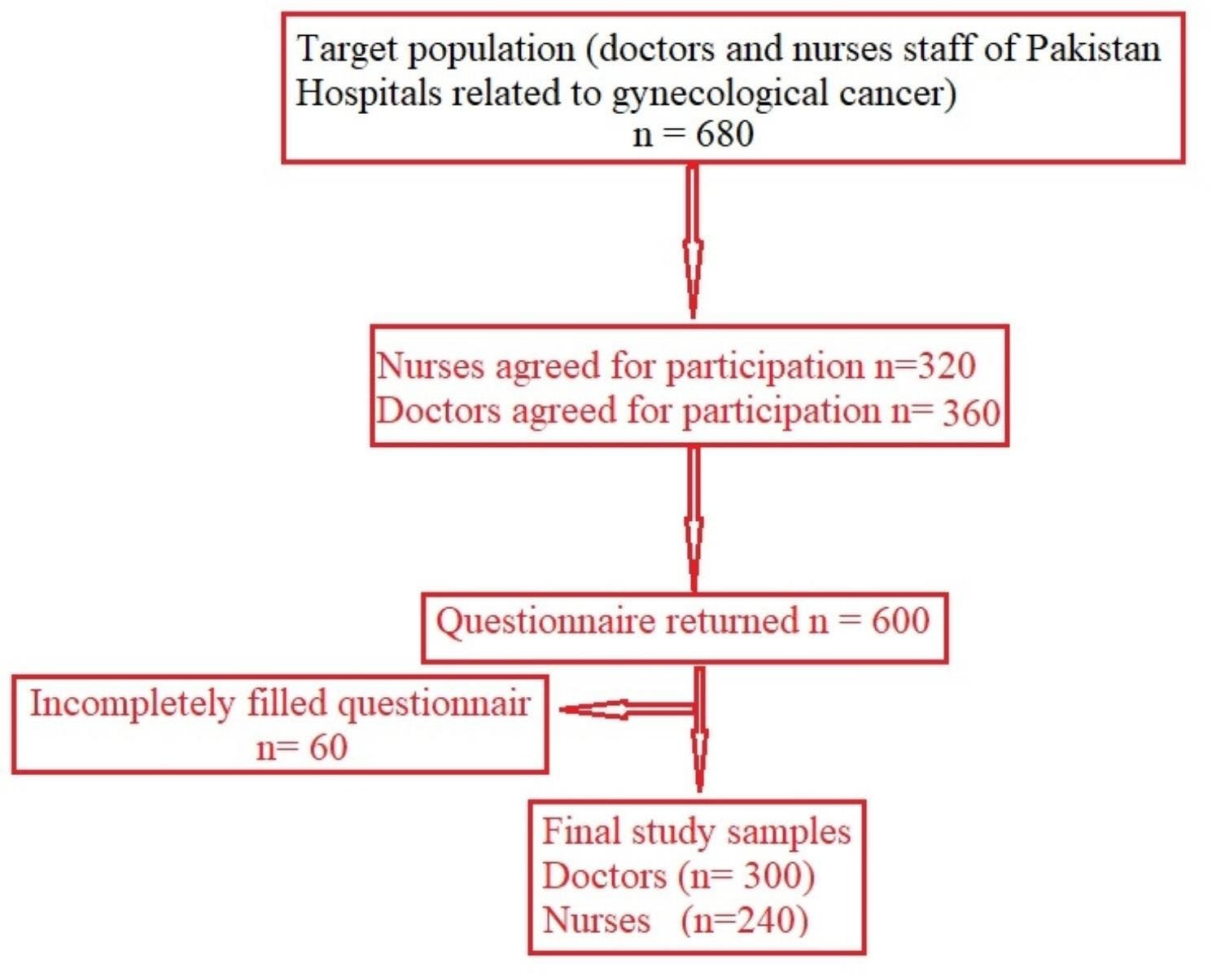
Study frame used in current study

Data was collected from the medical and nursing staff from gynecological wards of all the hospital of Pakistan. A total of 680 individuals including 320 nurses and 360 doctors were willing to participate in this study. Questionnaires (680) were sent to the participants and 600 questionnaires were returned. Among returned questionnaires, 60 questionnaires were incompletely filled and excluded from the study. The remaining 540 (nurses = 240 and doctors = 300) participants are included in the present study. The details study frame is given in Fig. 1.

Present study included the 240 nurses and 300 doctors from different hospitals of Pakistan. Data was collected by conducting the interviews and filling in the questionnaires. Particulars of nurses and doctors participating in present study are given in Table 1. Mean age of the nurses and doctors is 35 years, and 67% nurses have the age ≤ 35 years, and 33% nurses have the age > 35 years. In case of doctors, 19% have the age ≤ 35 years and 81% have the age > 35 years. In case of gender, 31% nurses are males and 69% are females and 15% doctors are males, and 85% doctors are females. With respect to marital status 81% nurses are married, and 19% nurses are unmarried. However, in case of doctors 69% are married and 31% are unmarried and difference in marital status between both respondent groups was found statically significant (p < 0.001). Participants are also questioned with respect to HPV vaccination, 39% have received the vaccination and 61% are found unvaccinated in case of nurses. 62% doctors were found vaccinated and 38% are unvaccinated and significant difference was observed between both group regarding HPV vaccination (p < 0.0001), as shown in Table 1.

**Table 1:** Particulars of participants of hospital care in cervical cancer patients

Sr no.	Particulars of participants		Nurses (n = 240)	Doctors (n = 300)	p-value
1	Gender	Males	74 (31)	45 (15%)	0.0001
		Females	166 (69)	255 (85)	
2	Religion	Islam	185 (77)	270 (90)	0.0004
		Others	55 (23)	30 (10)	
3	Age	< 35	161 (67)	58 (19)	0.0001
		>35	79 (33)	242 (81)	
4	Marital status	Married	194 (81)	206 (69)	0.001
		Unmarried	46 (19)	95 (31)	
5	Having a daughter	Yes	156 (65)	188 (63)	0.57
		No	84 (35)	112 (37)	
6	Economic status	Poor	89 (37)	34 (11)	0.0001
		Middle class	151 (63)	266 (89)	
7	Previously diagnosed	Yes	14 (6)	29 (10)	0.1
		No	226 (94)	271 (90)	
8	Vaccination with HPV	Yes	94 (39)	187 (62)	0.0001
		No	146 (61)	113 (38)	
9	Work experience	< 20 years	89 (37)	164 (55)	0.0004
		> 20 years	151 (63)	136 (45)	
10	Trained on screening	Yes	67 (28)	186 (62)	0.0001
		No	173 (72)	114 (38)	
11	Barriers observed during treatment	Shortage of staff	70 (29)	98 (33)	0.57
		Nurse gender	90 (38)	110 (36)	
		Shortage of trained nurses	80 (33)	92 (31)	

Analysis showed that 28% nurses have taken the training for screening of cervical cancer and 72% nurses were found untrained. However, in the case of doctors, 62% have taken the training for screening of cervical carcinoma and 38% were found untrained for screening. Further analysis showed the significant difference in nurses and doctors with respect to the screening training (p < 0.0001) as shown in Table 1. With respect to the barrier observed during the treatment of cervical cancer, both groups (nurses and doctors) of respondent have claimed that shortage of trained staff and nurses’ gender is the major barrier during the treatment of cervical cancer. Other details regarding the work experience and economic status of respondent are given in Table 1.

**Fig. 2:**
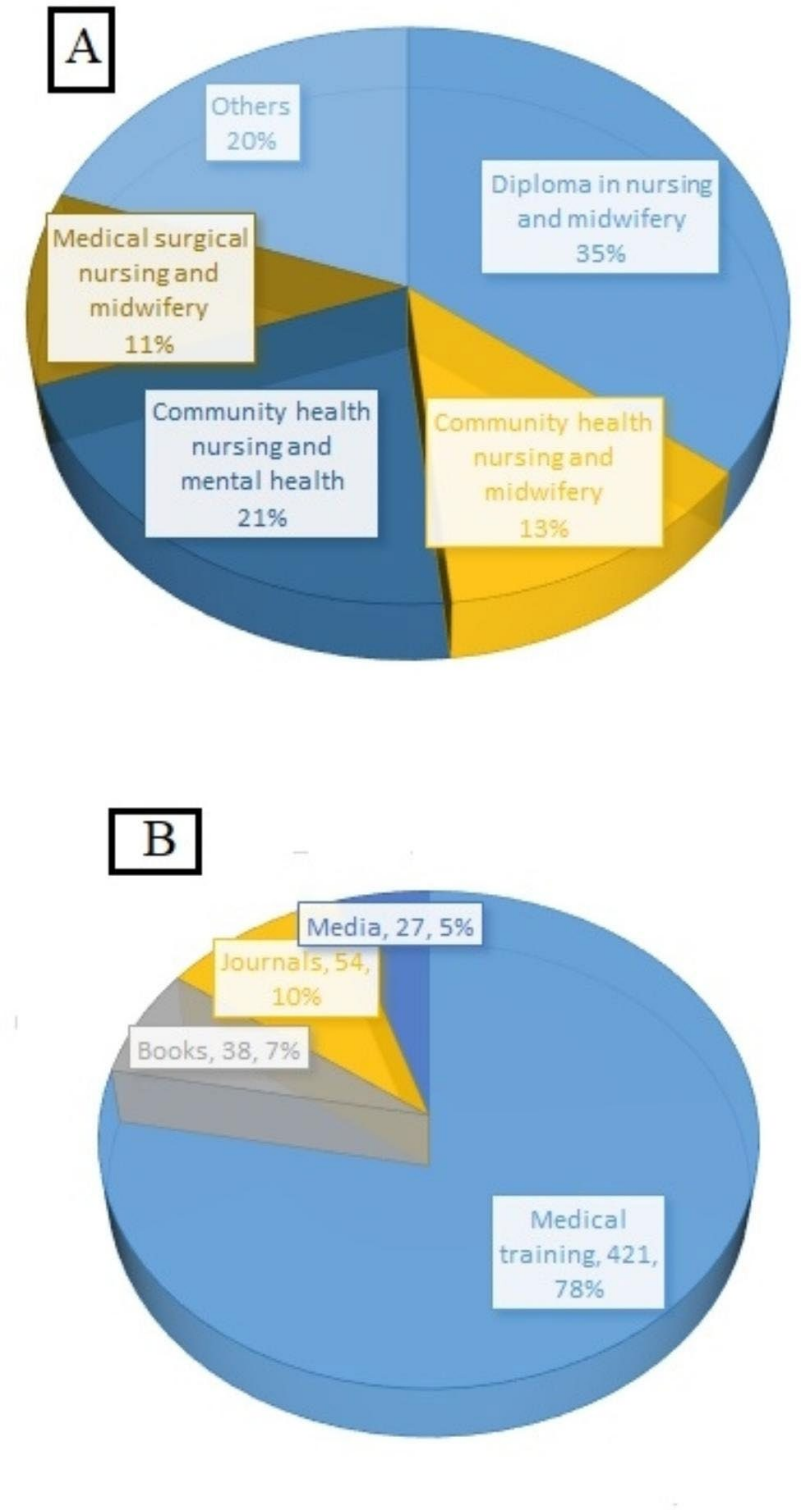
(A) Qualification of the nurses participating in present study cohort. (B) Source of knowledge used by nurses and doctors for cervical cancer care and treatment

In terms of nurse’s qualification, 77% nurses have received the diploma in different field of nursing and health care after basic education (middle and matric degree) and 23% have not received any qualification as shown in Fig. 2A. Data was also collected regarding the source of cervical cancer information and medical/nursing training was found to be the most common source of information for cervical carcinoma care and treatment as shown in Fig. 2B.

Experience of nursing/doctor staff during caring for cervical cancer patients is shown in Table 2. Among these parameters, 77% of patients feel helplessness due to terrible prognosis of the cervical cancer with respect to nurses’ opinion, and 63% patients feel helplessness with respect to doctors’ opinion. Significant difference was observed between both group respondent (p < 0.0002) as shown in Table 2. Data was also collected from both respondents with respect to suppose patient’s barrier, 44% nurses mentioned that lack of knowledge is barrier for treatment of cervical cancer. 70% doctors mentioned that cultural belief is the barrier for treatment and difference between nurses’ and doctors’ opinion was observed statistically significant (p < 0.0001), as shown in Table 2.

**Table 2:** Experiences of nursing and medical staff during care of cervical cancer patients

Sr no.	Experiences during care of Cervical cancer patients		Number (%)		P value
			Nurses (n = 240)	Doctors (n = 300)	
1	Supposed patient barriers	Patient fear	77 (32)	102 (34)	0.0001
		Cultural belief	89 (37)	210 (70)	
		Lack of knowledge	105 (44)	26 (9)	
2	Feeling helplessness due to a terrible prognosis	Yes	189 (77)	189 (63)	0.0002
		No	55 (23)	111 (37)	
3	Feeling of optimistic biasness	Yes	156 (65)	214 (71)	0.11
		No	84 (35)	86 (39)	
4	Prejudice of cervical cancer caused by sex	Yes	151 (63)	209 (70)	0.09
		No	89 (37)	91 (30)	
5	Having sympathy toward patients	Yes	113 (47)	234 (78)	0.0001
		No	127 (53)	66 (22)	
6	Are you aware of common psychological problems of patients	Yes	48 (20)	98 (33)	0.0009
		No	192 (80)	202 (67)	
7	Do you provide patients with psychological counseling	Yes	60 (25)	102 (34)	0.02
		No	180 (75)	198 (66)	
8	Are you having session of emotional guidance with patients	Yes	72 (30)	131 (45)	0.001
		No	168 (70)	169 (55)	
9	Caring experience for CC patients with invisible husband	Yes	82 (34)	106 (35)	0.77
		No	158 (66)	194 (65)	

In case of psychological assessment of patients, 80% nurses and 67% doctors do not have any knowledge regarding the psychological problems of the patients and difference between opinion of both respondents was observed statistically significant (p < 0.0009) as shown in Table 2. only 30% nurses and 66% doctors declared to provide the psychological counselling to the patients and difference between opinion of both respondents was observed statistically significant (p < 0.02). 63% nurses and 70% doctors believed that cervical cancer is caused by sex, however remaining believe on the other reasons as a causative factor of said diseases. Other parameters discussed with the respondent groups of cervical cancer such as caring experience for patients with invisible husband, feeling of optimistic biasness, having sympathy towards patients are given in Table 2.

In the present study, data was collected with respect to symptoms and risk factors of cervical cancer as shown in Table 3. Analysis showed that doctors have more knowledge regarding the symptoms of cervical cancer such as post-menopausal bleeding (p < 0.0001), menorrhagia (p < 0.0001), abdominal pain (p < 0.0001), post coital bleeding (p < 0.0001), fever (p < 0.0001), pelvic pain (p < 0.04) and weight loss (p < 0.03) compared to nurses as shown in Table 3. With respect to risk factors of cervical cancer, multiple sex partner, early age sexual intercourse and HPV infection history were considered as main risk factors for cervical cancer (Table 3).

**Table 3:** Knowledge about the Symptoms and risk factors of cervical carcinoma

Symptoms of cervical cancer		Nurse	Doctor	p-value
Post-menopausal bleeding	Yes No	168 (70) 72 (30)	256 (85) 44 (15)	0.0001
Menorrhagia	Yes No	54 (23) 186 (77)	210 (70) 90 (30)	0.0001
Foul-smelling discharge	Yes No	202 (84) 38 (16)	267 (89) 33 (11)	0.098
Abdominal pain	Yes No	160 (67) 80 (33)	67 (22) 233 (78)	0.0001
Post coital bleeding	Yes No	49 (20) 191 (80)	235 (78) 65 (22)	0.0001
Fever	Yes No	56 (23) 184 (77)	29 (10) 271 (90)	0.0001
Pelvic pain	Yes No	45 (19) 195 (81)	78 (26) 222 (74)	0.04
Weight loss	Yes No	67 (28) 173 (72)	60 (20) 240 (80)	0.03
**Knowledge regarding risk factors of cervical cancer**				
Smoking	Yes No	101 (42) 139 (58)	156 (52) 144 (48)	0.02
Multiple sex partners	Yes No	156 (65) 84 (35)	237 (79) 63 (21)	0.0002
Pruritis vulvae	Yes No	49 (20) 191 (80)	52 (17) 248 (83)	0.36
Sexual intercourse < 16 years	Yes No	210 (88) 30 (12)	264 (88) 36 (12)	0.86
Family history of cervical carcinoma	Yes No	195 (81) 45 (19)	259 (86) 41 (14)	0.11
Use of IUCD	Yes No	39 (16) 201 (84)	27 (9) 273 (91)	0.02
Poor hygiene	Yes No	25 (10) 215 (90)	32 (11) 268 (89)	0.93
Impaired Immunity	Yes No	34 (14) 206 (86)	26 (9) 274 (91)	0.04
History of HPV infection	Yes No	189 (79) 51 (21)	258 (86) 42 (14)	0.02

Table 4 showed the knowledge of nurses and doctors regarding the diagnostic/treatment modalities of cervical carcinoma. Pap smear test and cervical biopsy were considered as major diagnostic modalities according to opinion of the respondent group. Doctors have statistically more knowledge about the pap smear (p < 0.0001), cervical biopsy (p < 0.0001), colposcopy (p < 0.0001) and visual application after acetic acid application (p < 0.0001) compared to nurses’ knowledge for diagnostic modalities as shown in Table 4. Data was also collected with respect to screening and doctors have more knowledge regarding cervical cancer screening as compared to nurses. 92% doctors have opinion that females above the age of 30 years should be screened for cervical carcinoma, 90% doctors have opinion that unmarried females are also screened, and 75% doctors have opinion that females above the age 21 years should be screened for the cervical carcinoma as shown in Table 5. Data was also collected regarding the evaluation of diagnostic screening of cervical carcinoma. 87% doctors and 63% nurses’ respondents have the opinion that all women should be screened for the cervical carcinoma and difference between both opinions was found statistically significant (p < 0.0001) as shown in Table 5.

**Table 4:** Knowledge about the treatment modalities of cervical carcinoma

Treatment modalities		Nurses	Doctors	p-value
Have you ever heard about the diagnostic modalities of cervical carcinoma				
(i) Pap smear (ii) Cervical biopsy (iii) Colposcopy (iv) Visual application after acetic acid application	Yes No Yes No Yes No Yes No	159 (66) 81 (34) 132 (55) 108 (45) 29 (12) 211 (88) 24 (10) 216 (90)	260 (86) 40 (14) 267 (89) 33 (11) 210 (70) 90 (30) 243 (81) 57 (19)	0.0001 0.0001 0.0001 0.0001
Who should be screened?				
(i) Above 21 years (sexually active for last 3 years) (ii) Married women only (iii) Female above age of 30 years	Yes No Yes No Yes No	119 (49) 121 (51) 56 (23) 184 (77) 168 (70) 72 (30)	226 (75) 74 (25) 29 (10) 271 (90) 276 (92) 24 (8)	0.0001 0.0002 0.0001

**Table 5:** Detailed diagnostic evaluation of cervical carcinoma

Diagnostic evaluation of cervical carcinoma		Nurse	Doctor	p-value
Do you think all women should screening of cervical cancer	Yes No	152 (63) 88 (337)	262 (87) 38 (13)	0.0001
Is screening necessary after menopause	Yes No	176 (73) 64 (27)	195 (65) 105 (35)	0.03
Have you performed a pap smear screening	Yes No	132 (55) 108 (45)	261 (87) 39 (13)	0.0001
Have you ever got a pap smear screening	Yes No	59 (25) 181 (75)	126 (42) 174 (58)	0.0002
Cervical carcinoma can be detected even before symptoms appear	Yes No	65 (27) 175 (73)	121 (41) 179 (59)	0.001
Do you think you are at risk of getting cervical carcinoma	Yes No	109 (42) 131 (58)	162 (54) 138 (46)	0.04
If cervical changes are found early, are they curable	Yes No	211 (88) 29 (12)	263 (88) 37 (12)	0.9

In the case of pap smear screening, 87% doctors and 55% nurses have performed pap smear screening and screening frequency was observed significantly higher in doctors compared to nurses as shown in Table 5. Data was also collected for pap smear screening got by doctors and nurses. Frequency of Pap smear screening got by doctor (p < 0.0002) was observed significantly higher as compared to the pap smear screening got by nurses. 54% doctors and 42% nurses have the opinion of getting cervical carcinoma and approximately 88% nurses and doctors have the opinion that cervical carcinoma can be cured if diagnosed earlier as shown in Table 5.

Data was also collected to assess whether pap smear was performed in married females compared to unmarried females. Most of pap smear was performed in married females as compared to unmarried one. Difference between each group was observed statistically significant (p < 0.0001) as shown in Table 6. Analysis was also performed to check frequency of married and unmarried respondents who got the pap smear test. Results showed that married respondent who got pap smear screening was observed significant higher (p < 0.0001) as compared to unmarried respondents as shown in Table 6.

**Table 6:** Association between the marital status and Pap smear screening frequency in doctors and nurses

Marital status	Pap smear performed	Total	p value
	Pap smear performed Pap smear not performed		
Married Unmarried	278 45 115 102	323 217	0.0001
Marital status	Got a pap smear	Total	p value
	Pap smear taken Pap smear not taken		
Married Unmarried	167 232 18 123	399 141	0.0001

## Discussion

Cervical cancer is the second most common cancer in female after the breast cancer with high mortality rate. Around half of the million women diagnosed with cervical cancer and one quarter died due to this disease [28]. Cervical screening and disease awareness have significantly reduced the disease mortality rate in countries, where there is easy access to cervical cytology and proper nurse care to cervical cancer patients. So, proper training of nurses and doctors for cervical cancer patients is an important parameter to be address for the control of this deadly disease. The present study is designed to conduct interviews and to fill the questionnaires from nurses and doctors working in different hospitals for the care of cervical cancer patients. Data regarding the care of cervical cancer patients was collected from 240 nurses and 300 doctors and analyzed in the present study.

One of the major problems that needs to be address for the cervical cancer patients care is less qualification of the nurses. Most of the nurses are less educated with the simple diploma in nurses, community health nursing/midwifery, community health nurses/mental health and medical surgical nursing. Our data showed that perhaps, training and fellowships regarding the colposcopy, pap test and psychological counselling of cervical cancer patients are required to increase the survival rate, to better psychological/physical health of cervical cancer patients and ultimately to decrease the health burden due to this deadly disease. Mwaka et al., (2013) has reported that qualification of nurses is consider as the important challenge that can influence the nurses and health care decisions, management goals and practices towards the patients care and disease burden control [30]. Similarly, results have also been reported by previous studies [31].

Other important concern to lack of control of this disease is nurses and doctors feel helpless due to the terrible disease prognosis and increased death rate of the patients. This seriousness of disease became a barrier preventing the nurses and doctors from treating the patients positively. Previous studies have reported that cervical cancer increased incidence is due to lack of proper nurse’s care to patients due to fear of cervical cancer disease [32, 33]. Previous study has reported the nurse’s and doctor’s fear and feeling helplessness, as they considered it to be difficult to provide customized nurses care to meet patient’s difficult condition [34]. Andersen et al., (2015) has reported similar behavior of nurses and doctors towards the treatment/care of cervical cancer patients. Study stated that negative perception of cervical cancer act as the obstacle for the proper care of cervical cancer patients [35].

Our study also reported that HPV vaccination status of nurses and doctors is important to get rid the feeling of fear of professionals toward spread and prognosis of said disease. HPV vaccination is considered as primary prevention strategy for the cervical cancer [36]. High income countries have been successful in controlling the cervical cancer due to the integration of HPV vaccine into their routine vaccination plan along with some secondary preventive measures [36, 37]. This HPV vaccination plan may not be readily available in low-income countries due to limited infrastructure for health care delivery. Studies have showed that HPV vaccination is more effective in disease control if administered before the sexual activation at the age of 11 to 13 years and before HPV exposure [38, 39]. WHO have recommended that HPV vaccination should be integrated in the routine vaccination plan in each country to overcome the disease burden related to HPV. [40]. Health care professionals’ knowledge for the HP vaccination is very critical because these can educate the patients/people and ultimately results in increased vaccination rate and decrease in disease occurrence [38, 40]. Previous studies involving the health care professionals have reported that participant having the knowledge of cervical cancer and its prevention have a greater intentions to receive HPV vaccine [38–40]. Our data showed that 39% nurses and 62% doctors have received the vaccination and difference in vaccination frequency was observed statistically. Present study demonstrated 30% vaccination rate in nurses and the need to increase the vaccination rate of nurses to control the fear of cervical cancer spread. Williams et al. (2018) has reported that 9% nurses have awareness about the HPV vaccination and knowledge of HPV as risk factor for cervical cancer [29]. In other studies vaccination rate in Korean nurses has been reported 20.3% [41] and 34.2% [42].

Psychological counselling is required for better treatment and care of cervical cancer patients. Our study reported the lack of proper psychological support to cervical cancer patients which lead to more unstable behavior of patients and increased prognosis of disease. One of the reasons for increased psychological imbalance in patients is most nurses and doctors do not have knowledge of common psychological problems of patients, and they are unable to provide psychological counselling to patients. A present study showed that 20% of nurses and 33% provide psychological counselling to patients. Mwaka et al., (2013) has reported that physical and psychological care supports were most demanded by the cancer patients than any other support [30]. Similar result has already been reported by different studies [32].

In present study, 63% nurses and 70% doctors expressed the prejudice of cervical cancer caused by sex and remaining expressed the sympathy. This prejudice ultimately results in negative feelings towards the sexual partner of cervical cancer patients. Previous study has already reported the mixed trend of prejudice and sympathy of nurses/doctors towards the cervical cancer patients [34]. While other studies have not reported the prejudice against the cervical cancer patients in different cancer patients care studies [32, 43]. Nurses need to reduce prejudice and negative emotions while caring for cervical cancer patients. Participants have the need to emphasize the patient’s critical condition, patient care, and attempt to understand the needs of cervical cancer patients. Furthermore, our participants also showed that patients feel uncomfortable sharing their sexual life and emotional problem with the medical staff. Similar results have already reported in previous studies [28, 34]. Present study participants also reported the careless attitude of the patient’s sexual partner towards the patient’s care and treatment.

In the present study data was also collected from nurses and doctors with respect to treatment modalities used for cervical cancer. Pap smear, cervical biopsy, colposcopy, and visual application after acetic acid application were used as treatment modalities. In the present study 86% of doctors and 66% nurses have the knowledge of pap smear for treatment of cervical cancer patients. Our findings are similar to a study which showed that 79% medical professional have been aware of Pap smear test as screening of cervical carcinoma [44]. Another study has reported that 77% of respondents have the knowledge that pap smear can be used for the detection of cervical carcinoma [45]. Majid et al., (2022) has reported that 68.9% medical staff have the awareness of Pap smear as the screening test of cervical carcinoma patients in Pakistani population [46]. Most of doctors and nurses have the opinion that pap smear has been used only for the screening of married female instead of unmarried one in present study. Similar results have already been reported in India where married females are more likely been screened for pap smear as compared to unmarried females. Another study has also been reported that unmarried women normally refuse to screen themselves out of fear of the potential social stigma they would suffer if they had a test perceived to be used for sexually active women [47].

## Conclusion

The present study showed the complexities of healthcare professionals’ attitudes towards caring for cervical cancer patients. Nurses showed the lack of knowledge of disease, risk factors and feeling of prejudice and negative emotions to the patients and their sexual partner. Furthermore, present study also showed pap smear or other screening modalities are also used for screening of unmarried females or each sexually active patients, for proper diagnosis and management of cervical carcinoma.

## Future directions

Our study suggests training of the nurses and doctors with the development of educational plans should be implemented in each hospital. These plans should include theory driven, group-based training program/workshop, hands on workshop on cervical cancer screening procedure, lectures from the experts and availability of revised cervical cancer education materials. These education plans will help to educate the medical professionals for cervical cancer patients care while managing their disease fear, controlling their prejudice and negative emotions. Secondly proper psychological training/education such as offering of certain degree programs to doctors and nurses such as healthcare professionals and psychology degree programs should be planned for the medical professionals. This degree program will help to train healthcare professionals to understand the psychological state of cervical cancer patients. This will help in psychological counseling of the patients at the time of depression and stress. Third, an education plan should be designed for the patient’s family and sexual partner, which will help them to understand this complicated disease. These education plans should include the involvement of professional and religion societies to provide effective sexuality-related education, involvement of resident/family physician to provide patients care and support related education.

## Data Availability

All data generated or analyzed during this study are included in this published article.

## References

[1] Sung H, Ferlay J, Siegel RL, Laversanne M, Soerjomataram I, Jemal A (2021). Global cancer statistics 2020: GLOBOCAN estimates of incidence and mortality worldwide for 36 cancers in 185 countries. CA: Cancer J Clin.

[2] Zhang X, Zeng Q, Cai W, Ruan W (2021). Trends of cervical cancer at global, regional, and national level: data from the Global Burden of Disease study 2019. BMC Public Health.

[3] Sadia H, Shahwani IM, Bana KFM (2022). Risk factors of cervical cancer and role of primary healthcare providers regarding PAP smears counseling: case control study. Pakistan J Med Sci.

[4] Jradi H, Bawazir A (2019). Knowledge, attitudes, and practices among saudi women regarding cervical cancer, human papillomavirus (HPV) and corresponding vaccine. Vaccine.

[5] Hughes C (2009). Cervical cancer: prevention, diagnosis, treatment and nursing care. Nurs (Through 2013).

[6] Riaz L, Manazir S, Jawed F, Ali SA, Riaz R. Knowledge, perception, and prevention practices related to human papillomavirus-based cervical cancer and its socioeconomic correlates among women in Karachi, Pakistan. Cureus; 2020;12(3). 10.7759/cureus.7183 PMC7135727 32269867

[7] de Sanjose S, Quint WG, Alemany L (2010). Human papillomavirus genotype attribution in invasive cervical cancer: a retrospective cross-sectional worldwide study. Lancet Oncol.

[8] Gallego LS, Dominguez A, Parmar M. Human papilloma Virus Vaccine. StatPearls Publishing, Treasure Island (FL); 2022. 32965857

[9] Pathak P, Pajai S, Kesharwani H. A review on the Use of the HPV Vaccine in the Prevention of Cervical Cancer. Cureus. 2022; 14(9). 10.7759/cureus.28710 PMC9529156 36211088

[10] Farhath S, Vijaya PP, Mumtaj P (2013). Cervical cancer: is vaccination necessary in India?. Asian Pac J Cancer Prev.

[11] Chatterjee S, Chattopadhyay A, Samanta L, Panigrahi P (2016). HPV and cervical cancer epidemiology - current status of HPV vaccination in India. Asian Pac J Cancer Prev.

[12] Rosalik K, Tarney C, Han J. Human papilloma virus vaccination. Viruses. 2021;13. 10.3390/v13061091. 10.3390/v13061091 PMC8228159 34201028

[13] Hanson CM, Eckert L, Bloem P (2015). Gavi HPV programs: application to implementation. Vaccines (Basel).

[14] Bailey HH, Chuang LT, DuPont NC, Eng C, Foxhall LE, Merrill JK, Wollins DS, Blanke CD (2016). American society of clinical oncology statement: human papillomavirus vaccination for cancer prevention. J Clin Oncol.

[15] Abdallah AA, Hummeida ME, Elmula IMF (2016). Awareness and attitudes of nursing students towards prevention of cervical cancer. Cer Cancer.

[16] Ayinde OA, Omigbodun AO (2003). Knowledge, attitude and practices related to prevention of cancer of the cervix among female health workers in Ibadan. J Obstet Gynaecol.

[17] Daniyal M, Akhtar N, Ahmad S, Fatima U, Akram M, Asif HM (2015). Update knowledge on cervical cancer incidence and prevalence in Asia. Asian Pac J Cancer Prev.

[18] Owoeye IOG, Ibrahim IA (2013). Knowledge and attitude towards cervical cancer screening among female students and staff in a tertiary institution in the Niger Delta. Int J Med Biomedical Res.

[19] Parikh PM, Mullapally SK, Hingmire S, Uddin AK, Thinn MM, Shahi A, Tshomo U, Mohan I, Kaur S, Ghadyalpatil N (2023). Cervical Cancer in SAARC Countries. South Asian Journal of Cancer.

[20] Chughtai N, Perveen K, Gillani SR, Abbas A, Chunara R, Manji AA, Karani S, Noorali AA, Zakaria M, Shamsi U, Chishti U (2023). National cervical cancer burden estimation through systematic review and analysis of publicly available data in Pakistan. BMC Public Health.

[21] Batool SA, Sajjad S, Malik H (2017). Cervical cancer in Pakistan: a review. J Pak Med Assoc.

[22] Bowden SJ, Bodinier B, Kalliala I, Zuber V, Vuckovic D, Doulgeraki T, Whitaker MD, Wielscher M, Cartwright R, Tsilidis KK, Bennett P (2021). Genetic variation in cervical preinvasive and invasive disease: a genome-wide association study. Lancet Oncol.

[23] Tawe L, Choga WT, Paganotti GM, Bareng OT, Ntereke TD, Ramatlho P, Ditshwanelo D, Gaseitsiwe S, Kasvosve I, Ramogola-Masire D (2022). Orang’o OE. Genetic diversity in L1 ORF of human papillomavirus in women with cervical cancer with and without human immunodeficiency virus in Botswana and Kenya. BMC Infect Dis.

[24] Dardiotis E, Siokas V, Garas A, Paraskevaidis E, Kyrgiou M, Xiromerisiou G, Deligeoroglou E, Galazios G, Kontomanolis EN, Spandidos DA, Tsatsakis A (2018). Genetic variations in the SULF1 gene alter the risk of cervical cancer and precancerous lesions. Oncol Lett.

[25] SD A, Pasumarthi D, Pasha A, Doneti R, Botlagunta M, Pawar SC. Identification of differentially expressed genes in cervical cancer patients by comparative transcriptome analysis. Biomed Res Int. 2021 20;2021. 10.1155/2021/8810074 PMC8004372 33829064

[26] Liu W, Jiang Q, Sun C, Liu S, Zhao Z, Wu D (2022). Developing a 5-gene prognostic signature for cervical cancer by integrating mRNA and copy number variations. BMC Cancer.

[27] Roychowdhury A, Samadder S, Das P, Mazumder DI, Chatterjee A, Addya S, Mondal R, Roy A, Roychoudhury S, Panda CK (2020). Deregulation of H19 is associated with cervical carcinoma. Genomics.

[28] Abdallah AA, Hummeida ME, Elmula IMF (2016). Awareness and attitudes of nursing students towards prevention of cervical cancer. Cerv Cancer.

[29] Williams MS, Kenu E, Dzubey I, Dennis-Antwi JA, Fontaine K (2018). A qualitative study of cervical cancer and cervical cancer screening awareness among nurses in Ghana. Health Care Women Int.

[30] Mwaka AD, Wabinga HR, Mayanja-Kizza H (2013). Mind the gaps: a qualitative study of perceptions of healthcare professionals on challenges and proposed remedies for cervical cancer help-seeking in post conflict northern Uganda. BMC Fam Pract.

[31] Baqeas M, Rayan A (2018). Improving psychological well-being and quality of life among palliative care nurses: literature review. Am J Nurs.

[32] Lee MH, Lim EJ, Yu YH, Jun MH (2011). Clinical nurses’ HPV-related knowledge and perception of cancer causes: HPV vaccinated vs. not vaccinated. Korean J Women Health Nurs.

[33] Mawardika T, Afiyanti Y, Rahmah H (2019). Gynecological cancer inpatients need more supportive nursing care than outpatients: a comparative study. BMC Nurs.

[34] Schmid-Büchi S, Halfens RJ, Müller M, Dassen T, van den Borne B (2013). Factors associated with supportive care needs of patients under treatment for breast cancer. Eur J Oncol Nurs.

[35] Anderson NE, Kent B, Owens RG (2015). Experiencing patient death in clinical practice: nurses’ recollections of their earliest memorable patient death. Int J Nurs Stud.

[36] Ebu NI, Abotsi-Foli GE, Gakpo DF (2021). Nurses’ and midwives’ knowledge, attitudes, and acceptance regarding human papillomavirus vaccination in Ghana: a cross-sectional study. BMC Nurs.

[37] Charafeddine L, El Rafei R, Azizi S, Sinno D, Alamiddine K, Howson CP, Walani SR, Ammar W, Nassar A, Yunis K (2014). Improving awareness of preconception health among adolescents: experience of a school-based intervention in Lebanon. BMC Public Health.

[38] Sackey ME, Markey K, Grealish A (2022). Healthcare professional’s promotional strategies in improving human papillomavirus (HPV) vaccination uptake in adolescents: a systematic review. Vaccine.

[39] Biancarelli DL, Drainoni ML, Perkins RB (2020). Provider experience recommending HPV vaccination before age 11 years. J Pediatr.

[40] Collange F, Fressard L, Pulcini C, Sebbah R, Peretti-Watel P, Verger P (2016). General practitioners’ attitudes and behaviors toward HPV vaccination: a french national survey. Vaccine.

[41] Zeng Z, Huang Y, Li Y, Huang S, Wang J, Tang Y et al. Gene expression and prognosis of sirtuin family members in ovarian cancer. Medicine2020; 99(24). 10.1097/MD.0000000000020685 PMC7302638 32541517

[42] FA, Self-efficacy H, Belief. Influencing factors of efficacy, Health Beliefs, and knowledge of Cervical Cancer according to Vaccination or Presence of Cervical Cancer Among Nursing Students, 2016; 359–67.

[43] Mawardika T, Afiyanti Y, Rahmah H (2019). Gynecological cancer inpatients need more supportive nursing care than outpatients: a comparative study. BMC Nurs.

[44] Kim HW, Kim DH, Kim YH, Lee EJ, Kang SY, Lee DB et al. Clinical nurses’ awareness and caring experiences for patients with cervical cancer: a qualitative study. PLoS ONE; 14(5): e0217201. 10.1371/journal.pone.0217201 PMC6529155 31112578

[45] Iżycki D, Woźniak K, Iżycka N (2016). Consequences of gynecological cancer in patients and their partners from the sexual and psychological perspective. Prz Menopauzalny.

[46] Majid E, Shaikh MA, Qazi OA, Khan S, Majeed I, Bano K. Awareness, screening, Practices and attitudes of cervical cancer among doctors and nursing staff working at a tertiary care center. J Pak Med Assoc. 2022. 10.47391/JPMA.1443 35751302

[47] Shepherd MA, Gerend MA (2014). The blame game: cervical cancer, knowledge of its link to human papillomavirus and stigma. Psychol Health.

